# Patient experiences of nurse-facilitated advance care planning in a general practice setting: a qualitative study

**DOI:** 10.1186/s12904-019-0411-z

**Published:** 2019-03-06

**Authors:** Hilary Miller, Janice Tan, Josephine M. Clayton, Anne Meller, Oshana Hermiz, Nicholas Zwar, Joel Rhee

**Affiliations:** 1 School of Public Health and Community Medicine, University of New South Wales, Sydney, 2052 Australia; 2 Centre for Learning & Research in Palliative Care, HammondCare, Sydney, Australia; 3 Sydney Medical School, University of Sydney, Camperdown, Australia; 4 Advance Care Planning c/- Post-Acute Care Services, Prince of Wales Hospital, Randwick, Australia; 5 Center for Primary Health Care and Equity, University of New South Wales, Sydney, Australia; 6 School of Medicine, University of Wollongong, Wollongong, Australia; 7 Centre for Positive Ageing + Care, HammondCare, Sydney, Australia; 8 Faculty of Health Sciences and Medicine, Bond University, Gold Coast, Queensland Australia

**Keywords:** Advance care planning, Patient care, End of life, Patient reported outcome measures, Palliative care

## Abstract

**Background:**

Advance care planning (ACP) can offer benefits to patients and their families, especially when delivered in outpatient settings, but uptake remains low. Common barriers for health professionals include a perceived lack of time and adequate training, experience, and confidence in conducting ACP. Patient-reported barriers include a lack of awareness of ACP or discomfort initiating or engaging in discussions about end-of-life.

**Methods:**

We aimed to explore patients’ perspectives of an ACP intervention designed to address common barriers to uptake in the general practice setting. We provided training and support to doctors and general practice nurses (GPNs) to initiate and lead ACP discussions at their respective practices (2014 to 2015). Following the intervention, we conducted interviews with patients to explore their experience of engaging in ACP in the general practice setting. Thematic analysis was used to inductively code transcripts and identify key themes from semi-structured interviews with patients.

**Results:**

Six major themes relating to patient experiences of GPN-facilitated ACP were identified: working through ideas, therapeutic relationship with nurses, significance of making wishes known, protecting family from burden, autonomy in decision-making, and challenges of family communication. The patients valued the opportunity to speak about issues that are important to them with the GPN who they found to be compassionate and caring. The patients felt that ACP would lead to significant benefits not only to themselves but also for their family. Despite encouragement to involve other family members, most patients attended the ACP discussions alone or as a couple; many did not see the relevance of their family being involved in the discussions. Some patients felt uncomfortable or reluctant in communicating the results of their discussion with their family.

**Conclusions:**

With adequate training and support, GPNs are able to initiate and facilitate ACP conversations with patients. Their involvement in ACP can have significant benefits for patients. Psychosocial and relational elements of care are critical to patient satisfaction. Our findings show that some patients may feel uncomfortable or reluctant to communicate the results of their ACP discussions with their family. A future larger study is required to verify the findings of this pilot study.

## Background

Advance Care Planning (ACP) is a process through which patients may exercise personal autonomy in their wishes for end-of-life care, “to help ensure that people receive medical care that is consistent with their values, goals and preferences during serious and chronic illness” [1]. In Australia, end of life decisions are often made in acute care settings where patients may be unable to communicate their preferences and may have limited relationships with healthcare providers. In contrast, ACP conducted in the outpatient settings where patients are relatively stable can be a gradual and iterative process of decision-making and planning, with the potential to include carers and family members. Moreover, the ongoing relationships that clinicians in these settings have with their patients make them well placed to initiate and promote ACP [2].

ACP can confer significant benefits to patients and their families. Patients with advance-care-plans are more likely to have their wishes for end-of-life care known and respected [3]. ACP can improve quality of life in patients, and reduce stress, anxiety and depression in family members [3, 4]. Despite the benefits of ACP however, uptake remains low [5], particularly in outpatient settings [6].

Barriers to health professional initiating ACP include perceived lack of time, training or experience, low confidence, and discomfort with discussing end-of-life with patients [7–9]. Key barriers for patients include low awareness of ACP, discomfort relating to talking about end-of-life, fears that current treatment may be impacted, or perceptions that ACP is unnecessary in patients with good health [5, 6, 10, 11]. Having access to a clinician willing to initiate ACP could facilitate greater access [3].

These barriers apply to Australian general practice settings, especially the general practitioners (GPs) who are time-poor. In response, it has been suggested that General Practices Nurses (GPNs), nurses with training specific to the general practice setting, could address common barriers to accessing ACP and address patients’ psychosocial concerns. GPNs appear to be enthusiastic about their involvement in ACP. In a recent survey of general practice nurses conducted in New South Wales (NSW), 84% (*n* = 152) agreed or strongly agreed with the statement ‘Practice nurses should be involved in initiating and conducting ACP discussions with patients’ [12]. While there are many examples of nurses successfully completing ACP in varied settings such as intensive care and acute care settings [8, 9], there is lack of studies conducted to date that examined the feasibility and acceptability of general practice nurse involvement in ACP.

We have therefore designed and piloted an intervention to address common barriers to the uptake of ACP in general practice by training and supporting GPNs to conduct ACP with patients. In this paper, we report on the qualitative study that we conducted with these patients to understand how they experienced involvement in ACP in the general practice setting when common barriers to uptake were addressed, and what impact this had on patients and their families.

## Methods

### Setting

General practices form the cornerstone of primary health care delivery in Australia. Between 2014 and 2015, 83% of Australians had consulted a GP at least once in the previous 12 months [13]. The majority (83%) of GP consultations are fully funded by Medicare, a universal public insurance scheme. A typical general practice is a private small business, consisting of several GPs, GPNs, allied health professionals and administration staff. GPNs undertake a range of duties in the practice, but in recent years they have taken a greater role in chronic disease management and patient education [14].

### ACP and advance directives (AD) in Australia

Advance Directives (AD) is a medical order with legal protections to ensure it is followed. In Australia, laws relating to AD varies according to each State and Territory, with AD either being supported by statute law (determined by legislation), common law (determined by judges’ decisions from case law), or a combination of the two. Attaining an AD can be completed as part of ACP but is not a requirement nor synonymous with ACP. ACP is an ongoing and sometimes iterative process.

### Recruitment of practices

Four general practices in eastern Sydney took part in the study. To be eligible to participate, the practice needed to be: located in eastern Sydney; fully computerised; medium sized (3–8 GPs); have a significant elderly patient base; interested in implementing ACP; have a GPN who is willing to lead ACP; and have not taken previously part in a systematic approach to ACP. There were no other exclusion criteria for GPNs.

The practices were recruited with assistance from the local primary health organisation, which helped to send out invitation letters to GPNs, and promote the study to GP and GPN participants of an educational workshop on ACP. Invitation letters were followed up with a telephone call from the chief investigator. Participation of the GPNs and the GPs in the intervention was voluntary. Each practice was compensated for the time required for the GPN to attend the training and deliver the intervention to each patient. The GPNs were not compensated by the investigators for participating in the study beyond their existing employment wages/salaries.

### Education and training to practices

Five GPNs working in the practices received one-day training in ACP and implementation of the study. All the GPNs were Registered Nurses with a mean of 6 years experience in the general practice setting (range 2–13 years). None of the nurses had formal training in ACP or palliative care. The training workshop was supplemented with online modules containing videos of the recorded training sessions and other online resources. The educational resources covered: the practical aspects of discussing ACP with patients including the use of an ACP workbook and an Advance Care Directive template; communicating effectively regarding end-of-life issues; the legality of ACP in NSW; and the determination of decision-making capacity. The resources were developed by the investigators comprising of experts in ACP, palliative care, end-of-life communications, and primary care. A pre- and post-training knowledge questionnaire on ACP was administered to the GPNs and this assisted one of the investigators (AM), a clinical nurse consultant with expertise in ACP, to provide telephone support and mentoring throughout the study period. The participating GPs attended a brief educational session with a GP investigator (JR) on ACP. This session covered important aspects of ACP, especially issues that are likely to arise when completing and signing the ACP documents such as the legal validity of documents and assessing the capacity of the person signing legally-binding Advance Care Directives.

### Sampling of patient participants

Following the training, the GPNs, in conjunction with the GPs, identified patients who might benefit from ACP. In most cases, this was done opportunistically when the patient was visiting the GP or the GPN for another reason. To be included, a patient needed to be at least 18 years of age whom the GP and the GPN believed may benefit from ACP and able to provide informed consent. Practices recruited patients either opportunistically (e.g. asking them during a routine consultation) or systematically. The latter involved the practices reviewing the list of patients using the “Surprise Question” (i.e. the clinician asks the question “Would I be surprised if this patient was to die in the next 12 months?”) and/or the Supportive and Palliative Care Indicators Tool (SPICT) and sending out invitation letters by post to patients identified as potentially requiring supportive and/or palliative care. Exclusion criteria included patients under the age of 18 years of age or those unable to provide written informed consent. Patients fulfilling the inclusion criteria and not meeting the exclusion criteria were provided with written information and consent forms and invited to participate in the study.

### ACP intervention

The GPs of participating patients completed a referral form. This is a 3-page document developed by the investigators and designed to communicate the GP’s contextual understanding of the patient to the GPN coordinating the ACP discussions. It contains information such as the patient’s health condition, disease trajectory and their understanding of the patient’s social and family contexts.

The GPNs then conducted ACP sessions with the patients. More than one session could be booked for the discussions, depending on the need. Patients were encouraged to bring their family/substitute decision makers/caregivers to the discussions. An Advance Care Planning workbook and Advance Care Directive template was used to guide discussions and to record the patient’s wishes if required [15]. At the conclusion of the ACP sessions, especially if any Advance Care Plans or directives were completed, the GPNs arranged a consultation with the patient’s regular GP in order to review and sign the forms.

### Data collection

Basic demographic information of patient participants and the clinical information contained in the completed GP referral tools were collected at baseline.

At the conclusion of the intervention, all patients that participated were approached for an interview. One researcher (OH), with no prior relationship with the participants, conducted semi-structured phone interviews (average duration of 30 min). Interview questions explored patients’ previous experiences with ACP, perspectives on the intervention, the GPNs’ performance, and whether participating in ACP had impacted their families in any way (see Appendix A). Although patients were given the opportunity to have a support person present during the interviews, all patients opted to be interviewed alone. One patient chose to self-complete the interview guide and mail the response.

### Analysis

The patients’ baseline demographics and information contained in the completed GP referral tools were analysed using descriptive statistics. Interviews were audio recorded and transcribed verbatim by an independent transcribing service. All transcriptions and the single self-completed questionnaire were imported into Nvivo (QSR International, Version 10). Inductive thematic analysis was performed by a primary coder (HM). Line-by-line analysis was performed to extract salient themes and concepts. An initial coding tree was developed by HM and compared to an independent tree based on a subset of interviews developed by JT. Overall there was a high convergence of coding by HM and JT. Diverse codes were discussed within the research team and either incorporated or refined. The remaining transcripts were read by HM and JT. HM continued to code the remaining transcripts and to develop the coding tree and JT noted any emerging themes from transcripts they observed. The coding tree and emerging themes were refined in an iterative process following discussions with the research team (including a senior researcher (JR), and researcher/study interviewer (OH), and medical student (JT)). Thematic saturation occurred when no new concepts were emerging from the data.

### Funding source

The study was funded by a research grant from the Royal Australian College of General Practitioners/HCF Foundation.

### Ethics approval

The study received approval from the UNSW Human Research Ethics Committee (HC14305).

## Results

Of the 20 patients that received the ACP intervention, 13 participated in the interviews (70%). Most of the patients were recruited opportunistically. All patients attended at least one ACP visit with the GPN, lasting a mean of 32.2 min (10–75). The mean number of visits to discuss ACP was 2.4 (range 1–4). The GP was present during 35% of the first visit, 5% of the second visit, and 71% of the third visit, and 33% of the fourth visit.

Participants were aged 66 to 92 years (mean 81 years) and most were female (69%). While 42% were born outside of Australia, all spoke English at home. Around a quarter (23%) were married or in a de-facto relationship, half were widowed (46%), and 31% separated/divorced or never married.

The most common principal diagnosis was Ischaemic Heart Disease. Other diagnoses included other cardiovascular diseases, renal diseases, and cancer. Most patients had long-standing relationships with the medical practices; the average length of engagement was 14 years (ranging from 3 to 35 years). The frequency of visitation was also high, with an average of 23 visits in the last 12 months.

About a third of patients had arranged enduring guardians and almost none included family in the ACP discussions. Exceptions included two couples who attended together as part of a joint session but who did not invite other family members. Another patient was accompanied by her daughter on the initial visit but attended all remaining discussions alone. Two patients stated they were not offered the chance to invite family but felt they would not have involved family regardless had they been given the opportunity to do so.

We identified 6 major themes related to patients’ experiences of engaging in ACP with the GPNs: working through ideas, therapeutic relationship with nurses, significance of making wishes known, protecting family from burden, autonomy in decision-making, and challenges of family communication.

### Working through ideas

Overall patients found the discussions with nurses helpful in working through ideas around end-of-life care. While patients varied to the extent to which they had previously thought about their wishes, they felt nurses facilitated a deep consideration of their priorities and values, *“it made me think deeply about what was important to me” (Patient 10,101)*. Patients appreciated the interactive nature of the discussions as they felt comfortable to ask questions and raise concerns. Patients who had previously engaged in ACP reported that discussions with the GPN led to a greater consideration of issues, *“this was a deeper discussion and we covered topics that made me rethink a few things … it gave me more scope” (Patient 10,101)*. Most patients appreciated the flexibility around the number and duration of visits. As one patient reflected, “*we spent a bit of time doing it and it wasn’t rushed … when it’s the first time you sit down and do things like that you probably don’t have immediate answers*” *(Patient 30,201)*. However, patients’ preferences varied with respect to the depth, frequency and duration of discussions; one patient felt the discussions could have been enhanced by a broader discussion of the physical aspects of death and of spirituality, another patient felt discussions were at times vague, and two patients felt discussions could have been shorter in length. Overall, the discussions with nurses led most patients to reach a point of clarity and assurance about their wishes, “*I found it extremely helpful to have [the GPN] explain several matters in the questions and it helped to clarify my thoughts … with a bit of guidance from her I was able to crystallise what I wanted*” *(Patient 10,103)*.

### Therapeutic relationship with nurses

Patients reflected positively on the role of the GPNs, perceiving them to be knowledgeable, friendly, and helpful. Patients appreciated the openness and honesty of the nurses and their willingness to provide objective professional advice. As a result, patients felt comfortable to ask questions and speak openly, *“it was a great advantage to be able to speak in a confidential manner to the practice nurse. You share a lot of thoughts about end-of-life with each other without them trying to influence you. They just explain things, leaving you free to express your thoughts too” (Patient 10,101)*. The empathetic nature of the nurses was also noted, *“she allowed me to stop anytime I wished, if I found it upsetting to talk about things. She was very sympathetic and very helpful” (Patient 10,101)*. Patients drew contrasts between the therapeutic relationships with the nurses compared to previous experiences with GPs. Most felt the GPNs were more approachable and adept at dealing with psychosocial issues; “*I feel more comfortable with [the GPN]. I don’t really see the doctor very often, it’s only for prescriptions and such. I just found [the GPN] was probably more approachable” (Patient 40,203)*.

For some, satisfaction with the GPN discussions appeared to be moderated by existing relationships; one patient preferred that the discussions were with the GPN due to an existing relationship, and another patient was satisfied with the GPN but given their existing relationship with the GP would have preferred the discussion to be held with them. One patient stressed the importance of the respect and empathy of the health professional conducting the discussions, regardless of their role as a nurse or doctor. They felt the person conducting the discussions *required “the right personality, the right care and compassion” (Patient 20,103)*.

### Significance of making wishes known

Patients appreciated the opportunity to actively make decisions about their care, and to make these wishes known. They felt ACP had value in making their wishes known to family members. Patients also reflected on the benefits of ACP for individuals without living relatives or close family members, to guide health professionals should they be unable to communicate their wishes. The process of making one’s wishes known led to a sense of reassurance, *“if I had a stroke for instance and I couldn’t talk, I would be happy in the knowledge that my daughter knew exactly what I wanted” (Patient 20,103)*. Patients reflected on the benefit of having a physical document outlining their wishes*, “I felt comfortable having it written down to make sure that the family would know what my wishes would be” (Patient 30,201)*. Some patients felt documenting wishes was essential to provide clarity, especially given the unpredictability of health. As one patient noted, “*you never know what the world brings, it’s good to have something like this on paper” (Patient 40,202).*

Patients were fearful of physical and cognitive impairment. Documenting their wishes was seen as a way of safeguarding against unwanted medical procedures and ensuring their preferences would be respected by health professionals and family alike. One patient stated, *“my daughter-in-law kept saying, ‘You’ve got to get it down in writing before they’ll do anything about it’” (Patient 30,207)*. Another patient felt documentation would prevent family members from overriding their requests, *“they tend to interfere so it’s got to be clear to them what my wishes are” (Patient 40,202)*. However, for one patient there was a perceived limit to the power of the directive against family wishes, *“It made me realise that it doesn’t matter what I say really, it would depend on others … if one of my children or someone says, ‘we’re going to resuscitate him’, no matter what I’ve said, it’ll happen” (Patient 10,103)*. Most felt the timing of the ACP was appropriate for them, but a few felt it could have been held earlier. Knowing when to initiate ACP was acknowledged by patients as difficult to judge and highly personal. Influential factors included patients’ age and health status.

### Protecting family from burden

Patients felt ACP benefitted their family members by protecting them from the burden of making decisions on their behalf. By clearly outlining their wishes, patients felt they could prevent feelings of uncertainty and concern for family members, *“I think they would have made the same decisions in any case because they knew what I wanted, but I think that now they’ve got this piece of paper they don’t have to worry about it” (Patient 10,103)*. Patients felt this removed some of the weight of responsibility that family members might experience when making decisions. Some believed that having clarity on these issues provided a sense of reassurance to family, *“they seemed to be relieved that they’ll know what my wishes are” (Patient 10,104)*. Some also felt the ACP may protect family members from potential disagreement or conflict resulting from uncertainty. As exemplified by one patient, *“it’s like a will. In a will you leave whatever material things you have, you distribute it. Well, with your body you tell them what you want to do with your body … and then there’s no arguments” (Patient 30,201)*.

Many patients felt it was unnecessary to include family members in discussions with the GPN. They perceived this as a task they would complete independently, *“as far as I’m concerned it’s not them making decisions, it’s me” (Patient 10,103)*. Overall patients felt their families were understanding and comfortable with this, “*I think that they understand that I’ve been very independent for a long time and they respect my ideas and opinions, what I want to do with my life” (Patient 40,202)*. While some patients did not have close living relatives, the importance of autonomy was still highly valued, *“I don’t think I’m isolated or anything like that, but I usually do as much as I can on my own … I think it was best on my own” (Patient 40,201)*. There were a few exceptions; a few patients did not include family as they felt consultations would have been inconvenient or impractical due to the distance of living relatives, and other commitments. Two patients felt it would be emotionally burdensome for the family to attend, *“my sister...she’s got trouble on her own, never mind me to discuss my troubles with her” (Patient 20,105)*. One felt it was unnecessary to involve her family as she and her partner had already discussed their plans with their children and received advice from them, “*they were quite aware of what we were going to be discussing there … and also [my husband] he’s gone through a very bad stage, we didn’t know whether he’d live or die on two occasions, so I didn’t want to impose on them when I didn’t have to” (Patient 30,207)*.

### Challenges of family communication

Patients appeared to view communication with family as separate to the ACP process. While most did not include family members in the discussions with the GPN or GP, many of the patients communicated their decisions with the family themselves. However, the extent to which they discussed their plans with family varied significantly. Some patients reported being very open and direct throughout the process of ACP, “*as we were going along, we were telling my son and daughter-in-law about it … we’ve always been open about things, and they were aware of our preferences” (Patient 30,207)*. Others had less in-depth conversations with family, *“we didn’t go into it very thoroughly, I just told them what I had decided … that I agreed to become an organ donor and a couple of medical things that I didn’t want, that’s all” (Patient 10,104)*. A few patients did not openly discuss their wishes but provided copies of the ACP document to the family.

While overall patients felt comfortable in discussions with the nurses, most found talking to their family much more challenging. Some patients acknowledged their own reluctance talk about death, *“I’m pretty private about things like that” (Patient 30,201)*. Most patients acknowledged that family members could be reluctant to think or talk about death, “*They weren’t that keen on discussing it. They accepted what I told them, but they didn’t want to go into it very deeply … I suppose they didn’t like the thought of me not being here. Some people don’t like to talk about death or accept that it’s going to happen” (Patient 10,104).*

The extent to which patients discussed their wishes with different members of their family varied. For some, it was expected that certain family members would carry out the task of informing others. One patient had discussed her wishes with her sister, provided a copy of the ACP to her daughter but did not feel she could talk to her son at all stating, *“I showed my daughter the form that said I didn’t want resuscitation. My son doesn’t handle it too well, he gets emotional and tells me not to talk about it” (Patient 20,102)*. For some patients the ACP process highlighted the importance of communication and encouraged them to engage loved ones in conversations about end-of-life, *“it opened up a whole range of new ideas and discussions – like, I said to one friend, ‘Have you thought about it?’ and she said, ‘Oh, no, I’m not going to bother. My husband would know what I wanted.’ And I said, ‘I don’t think he would. He would probably think you would want to be revived and every care taken to keep you alive’” (Patient 10,101)*.

Openness to discussing end-of-life was influenced by the experience of the death of family members, and the reluctance of these family members to communicate their wishes,

*“I couldn’t talk to them about what they wanted at the end of their life and I didn’t want this to happen with me … I wish my mother had let me or started the conversation. I wish my brother had also, but they didn’t, and I didn’t feel that I should intrude upon their privacy by bringing the matter up. So, I’m really very happy to have this thing out in the open and discussed” (Patient 20101)*.

## Discussion

Our findings provide valuable insight into patient perspectives of ACP in the general practice setting, and the involvement of GPNs in the delivery of ACP. Patients were comfortable in discussions with nurses working within general practice and appreciated working through their ideas about end-of-life in a flexible and honest environment. The ACP process allowed them to exercise personal autonomy by making their wishes known, ultimately leading to a sense of reassurance. Patients also felt ACP benefited their families by reducing uncertainty and protecting them from the burdening of decision-making responsibilities; although the challenges of communicating one’s wishes with family were noted.

Many of our findings are consistent with other literature on patients’ perspectives on ACP. Reducing burden and conflict for family members through communicating wishes is often identified as an important motivation and outcome of ACP for patients [10, 11, 16, 17]. The literature also suggests that patients feel that making their wishes known could protect them from unwanted life-sustaining procedures, and from living with a physical or cognitive impairment [11], leading to patient reassurance [10, 11].

While some research indicates patients may be reluctant to engage with ACP due to expectations of fear and distress [17], we found that patients were comfortable during discussions. Nurses were perceived as honest, knowledgeable and approachable, and appropriate for dealing with psychosocial issues, including end-of-life care. Patients’ satisfaction with ACP appeared to be moderated by relational and psychosocial aspects of care; which had more precedence than the professional role of the facilitator as either a doctor or a nurse. This is consistent with research indicating the patient-clinician relationship is a key factor in patient satisfaction with ACP [17–19]. In line with this research, we found patients value honest, compassionate, and respectful communication [3], and prefer to engage in ACP with clinicians with whom they have existing relationships [3, 17, 20]. All in all, our findings support the nurses having a central role in ACP in the general practice setting.

Consistent with previous research [10, 17, 21], patients felt getting the timing right when initiating ACP was difficult, and recognised end-of-life as a taboo topic, with some acknowledging their own reluctance to talk about end-of-life or ACP. Patients preferred health professionals to initiate ACP discussions, rather than raise the issue themselves as found in previous studies [3]. Patients with a serious illness and those who had experienced the loss of a close family member were especially motivated to engage in ACP, as shown previously [11, 16, 19, 22].

While it was expected that patients would include family members in ACP discussions, almost no patients chose to do so. Most saw the discussions as something they would complete on their own. We speculate this may have been partly related to the location of discussions within medical practices in community settings; an environment in which patients tend to attend appointments alone and confidentiality is highly emphasised. Family engagement and communication varied widely between patients with some finding it easy to have these discussions, while others found it difficult. Consistent with previous research, these differences were related to factors including patient/family openness, acceptance of illness, family dynamics and physical distance of family members [19, 20]. Our findings also highlight the need to provide support to patients in having these discussions with the family members.

Preferences towards patient autonomy versus shared decision-making are varied in the literature [17, 19, 23]. Our findings also indicate that autonomy is critical to some patients, but others prefer a shared decision-making approach. In addition, there were some issues raised by patients that were not addressed in ACP including financial, legal and spiritual aspects of end-of-life. The variability in patient preferences for ACP demonstrates the need for ACP to be adaptable to patient preferences [11, 20]. Therefore, the clinician should be prepared for issues and questions going beyond the biomedical aspects of care and ensure appropriate referral to external supports are provided to ensure greater patient satisfaction [24].

Our findings may have been influenced by our sample, mostly comprising of long-term patients of the practices with chronic conditions but largely independent and in stable health. It is possible that the use of both an opportunistic approach to identifying patients in addition to a systematic approach in this study may have led to the selection of patients with these characteristics. In Addition, not all patients who received the ACP intervention took part in an interview. However, nearly two-thirds of the patients did participate in the interviews and our findings are mostly consistent with other qualitative research on this topic, lending support to the transferability of the findings.

## Conclusions

Our findings indicate that with adequate training and support, nurses working in general practice settings are able to initiate and facilitate ACP conversations with patients that result in positive patient outcomes. Nurse involvement in ACP can have significant benefits for patients, as they are able to clarify their preferences, make their wishes known, and reduce future burden for families. Psychosocial and relational elements of care are critical to patient satisfaction. Our findings show that ACP conversation can be a two-stage process for many patients; a discussion between themselves and health professionals, followed by a conversation with their family. There is a need to provide additional support to patients in having these discussions with their family. ACP should be flexible, guided by patient preferences, and allow for shared-decision making if appropriate. ACP delivered by GPNs has the potential to address barriers to uptake whilst maintaining patient satisfaction. A future large study with adequate power is required to verify the findings from this small pilot study.

## Abbreviations

ACP: Advance care planning
GP: General practitioner
GPN: General practice nurse
